# P-574. Characterization of N. meningitidis in Military Trainees and Implications for Vaccination Strategies

**DOI:** 10.1093/ofid/ofaf695.789

**Published:** 2026-01-11

**Authors:** Kevin Kashuba-Shanahan, Hui Xia, Erin Winkler, Theresa Casey, Ga On Jung, Angela Osuna, Lori Henrichs, Brian Casleton, John Kiley, Thomas Gibbons, Joseph Marcus

**Affiliations:** San Antonio Uniformed Services Health Education Consortium, San Antonio, TX; 59MDW/CIRS, San Antonio, Texas; San Antonio Uniformed Services Health Education Consortium, San Antonio, TX; BAMC, San Antonio, Texas; Defense Health Agency, San Antonio, Texas; BAMC, San Antonio, Texas; 59MDW/CIRS, San Antonio, Texas; Armed Services Blood Bank San Antonio, San Antonio, Texas; BAMC, San Antonio, Texas; 59 MDW/CIRS, Lackland AFB, Texas; Brooke Army Medical Center, San Antonio, TX

## Abstract

**Background:**

*N. meningitidis* is a gram-negative pathogen that is part of normal oropharyngeal flora, but can also cause meningitis outbreaks in susceptible populations, especially when encapsulated. The United States military vaccinates all entering trainees with a quadrivalent vaccine against serogroups A, C, W, and Y, but does not currently vaccinate against serogroup B. This study identified the predominant meningococcal serogroups (A, B, C, W, X, Y) in trainees upon arrival to BMT.

**Methods:**

Between June-August 2024, a convenience sample of 909 trainees received oropharyngeal swabs before meningococcal vaccination or penicillin prophylaxis. Swabs were cultured and subsequently serogrouped by polymerase chain reaction (PCR) as well as whole genome sequencing. For those that were PCR positive for serogroup B, the Meningococcal Antigen Typing System was used to determine coverage by the MenB-4C vaccines.

**Results:**

Of the 909 samples collected, 35 (3.9%) trainees were found to have oropharyngeal carriage of *N. meningitidis* and 4 (0.4%) were found to have oral infection with *N. gonorrhoeae*. Of the *N. meningitidis* isolated, 11 (31%) were able to be classified by serogroup by PCR with serogroup B accounting for the majority (72.7%) of groupable isolates. By whole genome sequencing, all *N. meningitidis* strains were found to be non-groupable, except for one isolate in serogroup C. The FDA approved serogroup B vaccine targets sub-capsular proteins, and not the capsule itself. Sequencing predicts that six (75%) of the serogroup B PCR positive samples would be targeted by the MenB-4C vaccine, with one being unpredictable, and one not covered.

**Conclusion:**

The vast majority of oropharyngeal isolates of *N. meningitidis* in a convenience sample of military trainees were non-groupable by whole genome sequencing despite having components of the capsule genes that are detectable by PCR. This data supports current practices of not vaccinating military trainees for serogroup B meningococcus.

**Disclosures:**

All Authors: No reported disclosures

